# Mechanical Injury Induces Brain Endothelial-Derived Microvesicle Release: Implications for Cerebral Vascular Injury during Traumatic Brain Injury

**DOI:** 10.3389/fncel.2016.00043

**Published:** 2016-02-29

**Authors:** Allison M. Andrews, Evan M. Lutton, Steven F. Merkel, Roshanak Razmpour, Servio H. Ramirez

**Affiliations:** ^1^Department of Pathology and Laboratory Medicine, Lewis Katz School of Medicine at Temple UniversityPhiladelphia, PA, USA; ^2^The Shriners Hospitals Pediatric Research CenterPhiladelphia, PA, USA; ^3^The Center for Substance Abuse Research, Lewis Katz School of Medicine at Temple UniversityPhiladelphia, PA, USA

**Keywords:** blood-brain barrier (BBB), traumatic brain injury, mechanical strain, extracellular microvesicles, tight junction proteins, neuroinflammation

## Abstract

It is well established that the endothelium responds to mechanical forces induced by changes in shear stress and strain. However, our understanding of vascular remodeling following traumatic brain injury (TBI) remains incomplete. Recently published studies have revealed that lung and umbilical endothelial cells produce extracellular microvesicles (eMVs), such as microparticles, in response to changes in mechanical forces (blood flow and mechanical injury). Yet, to date, no studies have shown whether brain endothelial cells produce eMVs following TBI. The brain endothelium is highly specialized and forms the blood-brain barrier (BBB), which regulates diffusion and transport of solutes into the brain. This specialization is largely due to the presence of tight junction proteins (TJPs) between neighboring endothelial cells. Following TBI, a breakdown in tight junction complexes at the BBB leads to increased permeability, which greatly contributes to the secondary phase of injury. We have therefore tested the hypothesis that brain endothelium responds to mechanical injury, by producing eMVs that contain brain endothelial proteins, specifically TJPs. In our study, primary human adult brain microvascular endothelial cells (BMVEC) were subjected to rapid mechanical injury to simulate the abrupt endothelial disruption that can occur in the primary injury phase of TBI. eMVs were isolated from the media following injury at 2, 6, 24, and 48 h. Western blot analysis of eMVs demonstrated a time-dependent increase in TJP occludin, PECAM-1 and ICAM-1 following mechanical injury. In addition, activation of ARF6, a small GTPase linked to extracellular vesicle production, was increased after injury. To confirm these results *in vivo*, mice were subjected to sham surgery or TBI and blood plasma was collected 24 h post-injury. Isolation and analysis of eMVs from blood plasma using cryo-EM and flow cytometry revealed elevated levels of vesicles containing occludin following brain trauma. These results indicate that following TBI, the cerebral endothelium undergoes vascular remodeling through shedding of eMVs containing TJPs and endothelial markers. The detection of this shedding potentially allows for a novel methodology for real-time monitoring of cerebral vascular health (remodeling), BBB status and neuroinflammation following a TBI event.

## Introduction

TBI represents a serious medical concern for patients and has both short-term (disorientation, memory loss etc.) and long-term (disability, loss of productivity etc.) effects. The pathology of TBI is due to a combination of primary (mechanical) and subsequent secondary (biochemical and cellular) effects. While previous TBI research has focused on damage to neurons, recent evidence has highlighted the central role of vascular integrity and changes in BBB permeability in delayed neuronal death and dysfunction (Shlosberg et al., 2010). In some cases, BBB dysfunction can last for months or years following the original injury (Korn et al., 2005). Prolonged BBB permeability can contribute to edema formation and result in cytotoxicity and further neuronal damage (Unterberg et al., 2004). Such observations are not only limited to cases of moderate or severe TBI, but can also be seen within the mild spectrum of TBI, including concussive and sub-concussive injuries. Part of its pathologic description commonly mentions the lack of radiological presentation of a hematoma, however this does not eliminate the possibility that the BBB has not been compromised. In fact, recent findings in mild TBI research have revealed the existence of micro-tears, which are small gaps or separations observed between endothelial cells, and sites of BBB breach (Glushakova et al., 2014; Liu et al., 2016). Thus, all forms of TBI at both the primary phase (induced by forces that are linear or rotational) and the secondary phase (a result of inflammation) trigger changes to the BBB, particularly at the level of single cell thick capillaries.

The brain endothelium is unique from other endothelium in that it has highly enriched levels of tight junction proteins (TJPs) such as occludin, claudin (Cld -3, -5 and -12), zonula occludens proteins (ZO-1, -2), and junctional adhesion molecules (JAM; Abbott et al., 2006). BBB integrity and formation of tight junctions (TJ) between endothelial cells (ECs) are vital for proper barrier function. However, our understanding of how mechanical injury and the secondary biochemical changes that occur in TBI affect TJs and TJPs remains unclear.

It is thought that all or most cells produce extracellular microvesicles (eMVs), which include microparticles and exosomes, in response to stimuli, activation, and injury. The release of eMVs has been shown to result from upstream signaling involving cytoskeletal remodeling and activation of GTPases RhoA and ARF6 (Boshans et al., 2000; Muralidharan-Chari et al., 2009; Latham et al., 2013; Ghossoub et al., 2014). Previous reports have shown that lung and umbilical ECs produce microparticles in response to mechanical stimuli (physiological shear stress and cyclic strain) *in vitro* (Vion et al., 2013a,b; Letsiou et al., 2015) and *in vivo* (Jenkins et al., 2013). In addition, an immortalized brain endothelial cell line (hCMEC/D3) has been shown to produce microparticles in response to cytokine and agonist stimulation (Latham et al., 2013). However, to date, no studies have shown whether brain ECs produce eMVs following TBI or whether eMVs contain TJPs that may reflect changes in BBB integrity. A recent study has shown elevated levels of the TJP occludin in the serum following a mild TBI (Shan et al., 2016); however, the eMV protein expression profile was never investigated.

To test whether TBI induces the release of brain endothelial-derived eMVs, we utilized an *in vitro* model that simulates tissue deformation caused by rapid changes in linear strain (such as that produced in TBI) applied to human brain endothelial cells (BMVECs). The model consists of BMVECs grown on elastic membranes that are deformed by vacuum to deliver a rapid injury. Our results indicate that the injury induces a time-dependent release of eMVs containing the TJP occludin. In addition, we detected elevated levels of endothelial markers in eMVs and increased endothelial activation of the small GTPase protein ARF6. Next, we confirmed the elevated release of occludin *in vivo* using the controlled cortical impact model of TBI. In addition, cryo-EM and immune-gold labeling of occludin demonstrated localization of occludin in eMVs from blood plasma following a TBI event. Overall, these results suggest that TJPs from cells that form the BBB are readily produced and detectable following mechanical injury to the endothelium. Importantly these findings also reveal the potential for using brain endothelial-derived eMVs as biosignatures for monitoring the health of the BBB not only during brain injury but possibly for other neurological conditions as well.

## Materials and methods

### Reagents

Rat-tail collagen I and endothelial cell growth supplement (ECGS) were purchased from BD Biosciences (Franklin Lakes, NJ). Heparin and bovine serum albumin (Sigma), DMEM/Ham's F12 media, Fetal bovine serum (FBS; Thermo Fisher Scientific, Waltham, MA).

### Endothelial cell culture

Primary human adult brain microvascular endothelial cells (BMVECs) were provided by Michael Bernas and Dr. Marlys Witte (University of Arizona, Tucson). BMVECs were isolated from vessels from the brain resection path of patients (showing no abnormalities) undergoing surgery for the treatment of intractable epilepsy, as previously described (Bernas et al., 2010). Isolation of cells and tissue and the use of BMVECs has been approved by Temple University and the University of Arizona's IRB committees. BMVECs were grown in Dulbecco's modified Eagle's medium/F12 media supplemented with 10% heat-inactivated fetal bovine serum (FBS; Thermo Fisher Scientific), endothelial cell growth supplement (ECGS; BD Bioscience), heparin (1 mg/mL; Sigma/Aldrich Co, Ltd), amphotericin B (2.5 μg/mL; Thermo Fisher Scientific), penicillin (100 U/mL; Thermo Fisher Scientific), and streptomycin (100 μg/mL; Thermo Fisher Scientific).

### *In vitro* mechanical strain-induced injury model

BMVECs were plated on Collagen I coated 6-well Flexcell plates (Flexcell International Corporation) and grown until confluence. The day before all experiments, media was exchanged with 10% FBS in DMEM F12 and then all experiments were conducted in 1% FBS DMEM F12. Endothelial cells were rapidly injured using a 1 s gradient biaxial strain (12 or 22% substrate elongation) exerted by vacuum application to the chamber using the Flexcell Tension System FX-5000 (Flexcell International Corporation). Percentage of Elongation is defined by the following equation:

$$\begin{array}{l} \%Elongation=\frac{L_{final}-L_{initial}}{L_{initial}}*100 \end{array}$$

Where *L* is the length of the substrate. The elongation of the membrane is a function of the applied force (from the vacuum) and the elasticity of the membrane. The following equation describes the force applied based on the vacuum pressure exerted:

$$\begin{array}{l} F=0.177*P*D^{2} \end{array}$$

Where *F* is the force applied in lbs, *P* is the pressure in MPa and *D* is the diameter of the sample in mm. The required force to achieve the desired % elongation is pre-programed by the manufacturer based on specifications for the Flexcell plates. Following injury, cells and media were harvested at time points (5 min and 2, 6, 24, 48 h) and processed for further analysis. For eMV harvest, media was spun at 2000 × g for 20 min to remove cellular debris and the supernatant was snap frozen and stored at −80°C. ARF6 activation was determined using a G-LISA assay (Cytoskeleton Inc., Denver, CO) following the manufacturer's protocol. A constitutively active ARF6 protein provided with the assay was used as a positive control.

### Live cell imaging and immunocytochemistry

For visualization of live cells after injury, cells were incubated with 1 μM of the cell tracker dye, Calcein-AM (Thermo Fisher Scientific) for 20 min prior to injury. Images were taken prior to and 2 h post injury. For immunocytochemistry, cells were fixed with 4% paraformaldehyde at 2 h post injury. The cells were then permeabilized with 0.1% triton for 10 min and then non-specific binding was blocked using 5% BSA for 30 min. Incubation of cells with primary antibodies to ZO-1 (BD Biosciences) was then performed overnight at 4°C. After rinsing at least three times with 1x PBS the cells were incubated with species specific Alexa-488 conjugated secondary antibodies for 1 h at RT (Thermo Fisher Scientific), rhodamine conjugated phalloidin for 1 h at RT (Cytoskeleton, Inc.) followed by counterstaining with nuclear dye DAPI for 30 min. Cells were then rinsed three times in 1x PBS and mounted in Prolong Antifade (Thermo Fisher Scientific) prior to coverslipping. All images were captured using a Coolsnap EZ CCD camera (Photometrics, Tucson, AZ) coupled to a Nikon 80i Eclipse (Nikon, Japan) and processed using the NIS Elements imaging software (Nikon).

### Flow cytometry of BMVECs

Metabolic activity assay: metabolic activity was determined using the dye C_12_-resazurin, which converts to red-fluorescent C_12_-resorufin in metabolically active cells (Thermo Fisher Scientific). Cells were rinsed with 1x PBS and then trypsinized. C_12_-resazurin was added (to a final concentration of 500 nM) and cells were incubated for 15 min at 37°C. Cells were washed and placed on ice and the fluorescence output was measured immediately. Cell Death Assay: % of dead cells was determined using STOX® Green, a green-fluorescent nuclear and chromosome counterstain that is impermeant to live cells (Thermo Fisher Scientific). Detached cells were pelleted from the media and combined with adherent cells, which were removed by trypsinization. STOX® Green was added to a final concentration of 10 nM and cells were incubated for 15 min at 37°C. Cells were then washed, placed on ice and the fluorescence output was measured immediately. Surface expression immunostaining of ICAM-1: Cells were rinsed with calcium and magnesium free 1x PBS, trypsinized and then pelleted by centrifugation at 1000 rpm for 5 min. Cells were then resuspended in fixation buffer (eBioscience Inc., San Diego, CA) and incubated for 10 min. Following fixation, cells were washed with flow cytometry buffer (eBioscience) and centrifuged as above. Cells were resuspended in 100 μL of flow cytometry buffer with an ICAM-1 preconjugated antibody to R-phycoerythrin (PE; eBioscience) for 1 h. Cells were then rinsed, centrifuged and resuspended in flow cytometry buffer for flow cytometry analysis. Data was acquired with a FACS BD Canto II flow cytometer (BD Biosciences) and analyzed using the FlowJo software (Tree Star, Ashland, OR, USA).

### Western blot of eMVs

eMVs were isolated using the Exoquick-TC system from System Biosciences Inc. (Mountain View, CA) following the manufacturer recommendation. eMVs were isolated from equal media volumes produced from equal cell numbers in order to control for differences in quantities of eMVs produced between conditions. All cell counts were obtained using an automated cell counter (Scepter, EMD Millipore, Billerica MA). The eMVs were then centrifuged and resuspended in Cell Lytic MT Cell Lysis Reagent (Sigma). Lysed eMVs were then mixed with 4x Laemmli buffer and boiled for 5 min at 95°C. Samples were loaded onto a 4–20% Mini-Protean TGX gel (Biorad, Hercules, CA). Gels were transferred to nitrocellulose membranes using the Trans-blot Turbo™ transfer system (Biorad) following the manufacturer's protocol. Membranes were blocked with 5% nonfat dry milk in PBST (0.5%) and all primary and secondary antibodies were resuspended in 5% nonfat dry milk in PBST (0.5%). Primary and secondary antibodies used were as follows: occludin (abcam, Cambridge, UK, rabbit, 1:1000), PECAM-1 (Santa Cruz Biotechnology Inc., Santa Cruz, CA, goat 1:1000), ICAM (Santa Cruz Biotechnology Inc., rabbit 1:1000), goat-HRP (Jackson ImmunoResearch 1:10,000) rabbit-HRP (GE Healthcare, Princeton, NJ, 1:10,000). HRP-conjugated antibodies were detected using Supersignal West Pico chemiluminescent substrate (Thermo Fisher Scientific) and visualization of luminescent signal was obtained using the gel documentation system, G:Box Chemi HR16 (Syngene, Frederick, MD). Densitometry was performed with the image analysis software, Image J 1.48v (NIH).

### Controlled cortical impact (CCI) mouse model of traumatic brain injury

The Institutional Animal Care and Use Committee (IACUC) at Temple University (Philadelphia, PA) approved all procedures detailed in this section that required the use of vertebrate animals prior to initiating any experimental objectives. Animals were weighed prior to surgery to obtain a baseline for monitoring animal wellness following CCI procedures. Animals were anesthetized using a 1:2 solution of ketamine (100 mg/ml) to xylazine (10 mg/ml; Henry Schein Animal Health; Dublin, OH) administered by intraperitoneal (IP) injection at a dose of 1 ml/kg. Depth of anesthesia was monitored throughout the surgical procedure by hind paw toe pinches to assure that animals remained properly anesthetized. Animals were shaved to remove hair from the scalp surrounding the surgical area and immobilized using a Mouse™ Stereotaxic Instrument (Stoelting Co.; Wood Dale, IL). Ophthalmic ointment (Dechra Veterinary Products; Overland Park, KS) was applied to the eyes to prevent ocular drying. Seventy percent isopropyl alcohol was used to clean the scalp, neck, and ears. A Zeiss Stemi 2000-C stereomicroscope (Carl Zeiss Microscopy, LLC; Thornwood, NY) equipped with a Schott EasyLED Ringlight (SCHOTT North America Inc.; Elmsford, NY) was used to magnify and illuminate the surgical site. Surgical instruments were autoclaved prior to use and sterilized between animals using a Hot Bead Sterilizer (Fine Science Tools Inc.; Foster City, CA). Surgical scissors were used to remove a portion of the scalp and expose the skull from the sagittal suture to the right temporalis muscle. The underlying fascia was removed, and an Ideal Micro-Drill™ (CellPoint Scientific Inc.; Gaithersburg, MD) with a 0.5 mm, rounded burr was used to create a 4 mm craniotomy between bregma and lambda suture lines. The surgical area was periodically washed with 1X PBS, and drill time was minimized in order to avoid overheating of surrounding tissue. The bone fragment resulting from the craniotomy was carefully lifted away from the underlying brain to avoid disrupting the dura and cortical tissue. Any animal sustaining additional injury to the brain during craniotomy procedures was excluded from the study. An Impact One™ Stereotaxic CCI Instrument (Leica Microsystems; Buffalo Grove, IL) outfitted with a piston (2 mm diameter) was secured to the stereotaxic stage and positioned over the exposed parietal somatosensory cortex. The piston was oriented parallel to the cortical plane and lowered until contact was made with the dural surface. TBI was delivered with the following parameters: impact velocity: 5 m/s, impact depth: 1 mm, impactor tip diameter: 2 mm, dwell time: 0.50 s. After discharging the impactor piston, the site of injury was covered by a sterile, 5 mm glass coverslip (Electron Microscopy Sciences; Hatfield, PA), which was secured to the skull using Vetbond™ tissue adhesive (3 M; St. Paul, MN), creating a waterproof seal between the glass coverslip and the surgical margins of the scalp. After adhering the coverslip atop the craniotomy, animals were removed from the stereotaxic stage and place in their home cage, resting upon an isothermal pad (Braintree Scientific, Inc.; Braintree, MA) to maintain body temperature during recovery. Animals were monitored in their home cage until consciousness was regained. Surgical control (craniotomy only) procedures included all of the steps described above except impactor discharge. Blood was collected from the inferior vena cava into an insulin syringe containing 20 μl of 0.5 M EDTA to prevent clotting. Whole blood was spun at 1500 × g for 20 min and plasma was transferred to a sterile microcentrifuge tube, snap frozen, and stored at −80°C for later use.

### Flow cytometry of eMVs from blood plasma

eMVs were isolated using the Exoquick system (System Biosystems, Inc.) following the manufacturer's protocol. In short, blood plasma was incubated with Exoquick for 30 min at 4°C. After isolation, eMVs were centrifuged at 1500 × g for 30 min and resuspended in 100 μL of flow cytometry buffer with primary antibody Occludin (abcam) for 1 h. FITC conjugated Secondary antibody (eBioscience) was added for 30 min. 200 μL of flow cytometry buffer was added and the samples were analyzed by flow cytometry. Events were acquired for each sample with a FACS BD Canto II flow cytometer (BD Biosciences) and analyzed with FlowJo software (Tree Star, Ashland, OR, USA).

### Cryo-EM of eMVs from blood plasma post-TBI

Isolated eMVs were resuspended in 100 μL of buffer containing 20 mM Tris pH 7.4 and 150 mM Na/KCl. eMVs were incubated with rabbit anti-occludin antibody (abcam) for 1 h followed by incubation of anti-rabbit IgG (H&L) conjugated to 10 nm gold nanoparticles (Cytodiagnostics Inc., Ontario, Canada) for 30 min. eMVs were prepared by high pressure freezing (or cryo-frozen) and analyzed by electron microscopy (EM) at the Electron Microscopy Resource Laboratory (EMRL) headed by Dr. Dewight Williams with technical support by Ray Meade. The EMRL is part of the University of Pennsylvania Biomedical Research Core Facilities.

### Statistical analysis

The experiments were independently performed multiple times (at least three times for all the data shown) to allow statistical analyses. Within each individual experimental set, every condition was evaluated in three replicates. The data collected were analyzed using Prism v6.0c (GraphPad Software, San Diego, CA). Multiple group comparisons were performed by two-way analysis of variance (ANOVA) with post-hoc analysis or students *t*-test where indicated. All the results are expressed as the mean ± SEM with differences considered significant at *p* < 0.05.

## Results

### Characterization of brain endothelial status following an *in vitro* model of mechanical injury

**Figure 1:**
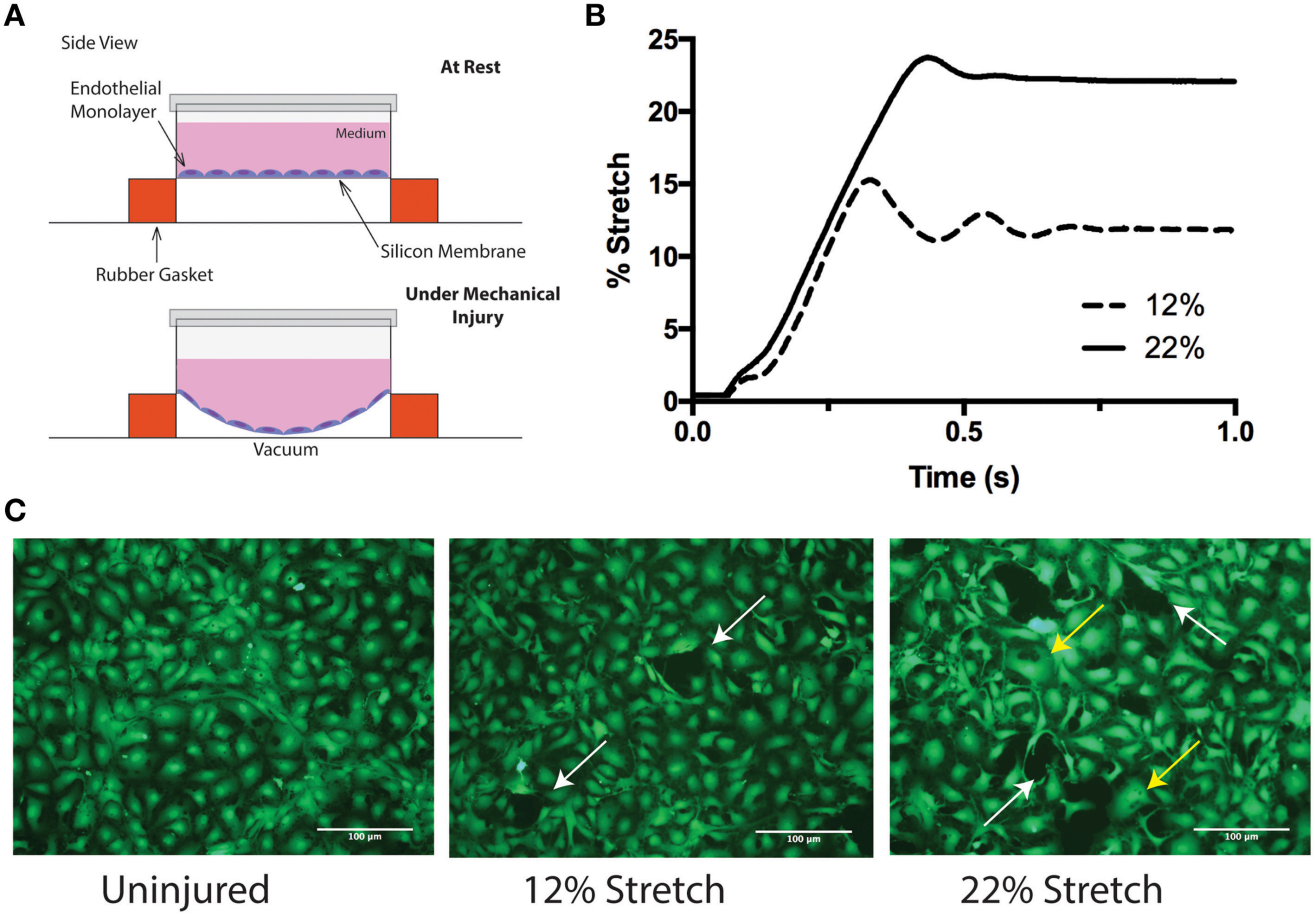
**An *in vitro* model of inducing mechanical biaxial strain. (A)** Schematic of the Flexcell™ injury device. Endothelial cells are grown on elastic silicone membranes that are deformed using negative pressure. **(B)** Stretch profile of the injury model. Two stretch parameters were used: 12 and 22% membrane elongation. Injury was performed by applying vacuum over 1 s. **(C)** Calcein-AM labeling of endothelial cells before and after injury. Uninjured cells are confluent and continuous with neighboring endothelial cells (left). Following 12% (middle) and 22% (right) stretch, miro-tears between adjacent endothelial cells develop (white arrows) and at the higher elongation, some cells appear to be swollen (yellow arrows).

To model the type of linear kinetic forces that lead to stretching-strain deformation of tissue during the primary phase of TBI, a Flexcell® Tension System FX-5000 (from Flexcell International Corp.) was utilized. This approach tested the ability of mature monolayers of primary human brain microvascular endothelial cells (BMVECs) to sustain rapid changes in mechanical strain for which micro-tears would be the observable outcome. In addition, this model utilizes a single layer of BMVECs and mimics capillary walls, which are likely key sites of vulnerability in mechanical injury during TBI. Similarly to physiological conditions, mature BMVECs (as described previously) in the above culture conditions display all normal BBB characteristics. Therefore, BMVECs grown on collagen type I coated silicone membranes were subjected to biaxial strain generated by vacuum pump negative pressure, (Figure 1A schematic of cells at rest, top, and under strain, bottom). Figure 1B shows the degree of deformation (membrane elongation profile) when mechanical stimulation was initiated by a semi-sinusoidal tensile stretch pulse lasting 1 s. Profiles are shown for stimulation frequencies of 1.0 and 0.5 Hz, corresponding to membrane elongation of 12 and 22% respectively. Next, the BMVECs were assayed for the presence of micro-tears after mechanical insult. For better visualization of BMVECs, the cells were loaded with the cell tracker Calcein-AM, which labels and allows the cells to be imaged by fluorescence microscopy. A continuous BMVEC monolayer can be seen in the undisturbed or uninjured control (**Figure 3A**). In contrast, both the 12 and 22% elongation increased micro-tear formation between adjacent cells (white arrows). In addition, an increased number of swollen or enlarged cells can readily be seen following the 22% elongation mechanical injury (yellow arrows). Taken together, these results confirm that strain deformation at either 12 or 22% elongation induces marked effects to brain endothelial cells in the form of micro-tears and cellular swelling.

### Mechanical injury affects intercellular tight junctions (TJs)

**Figure 2:**
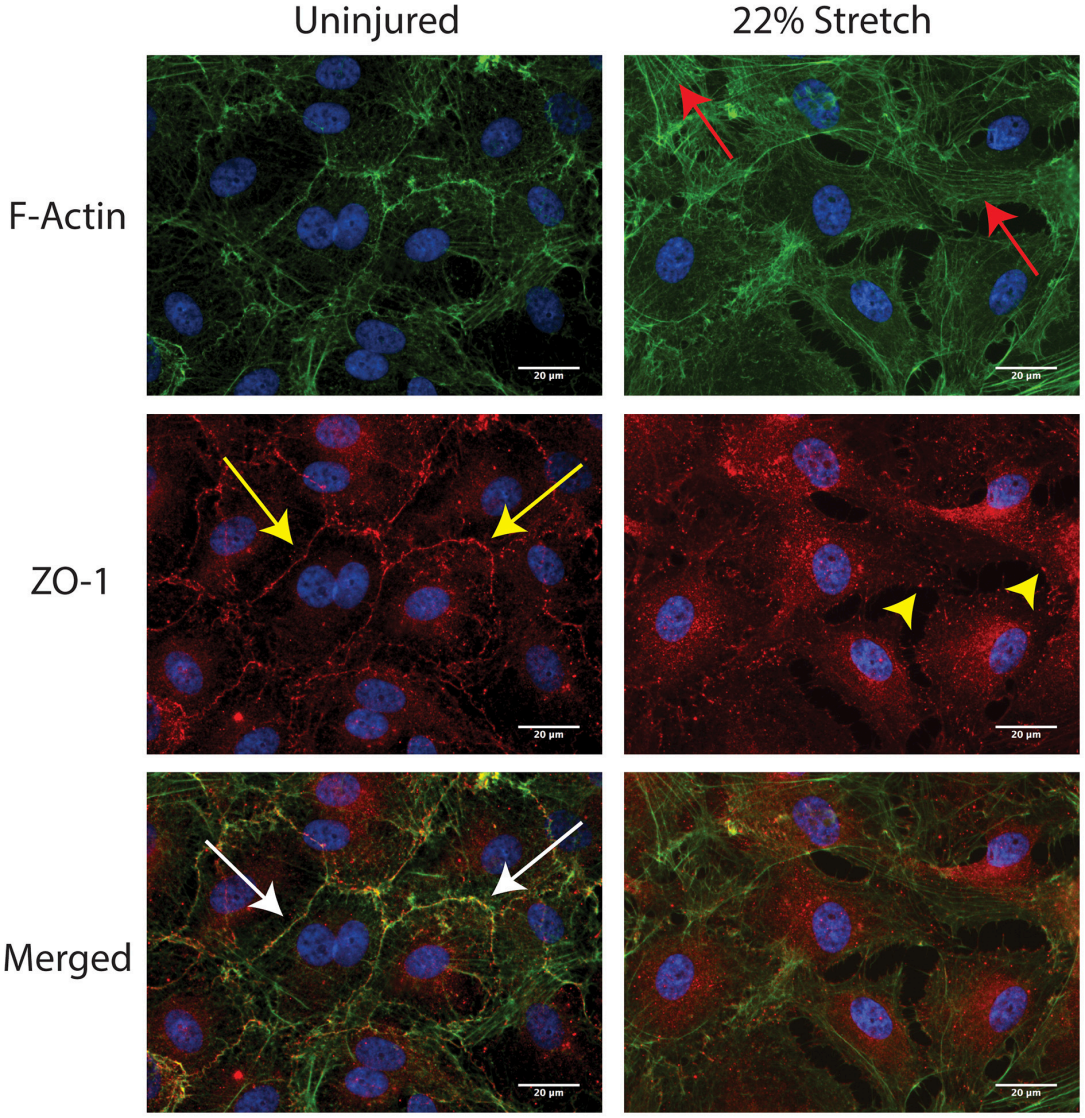
***In vitro* injury to BMVECs induces F-actin stress fiber formation and disruption of tight junctions**. Staining of uninjured BMVECs (left panels) shows actin localized primarily at the cell borders where it colocalizes with the tight junction protein ZO-1 (white arrows). In addition, minimal stress fibers along the length of the cell are visible and ZO-1 staining localized at the cell borders (yellow arrows) is typical of that seen in barrier forming BMVECs. Following injury by 22% membrane stretch (right panels), BMVECs develop clear and distinct F-actin stress fibers (red arrows) and ZO-1 redistributes to a punctate pattern at cell borders with diffuse expression across the cell (yellow arrowheads).

The physical manifestation of the BBB is due to the presence of tight junction proteins (TJPs) between neighboring endothelial cells (Persidsky et al., 2006). Tearing or dysfunction of the BBB in essence is suggestive of disconnected and disassembled TJs (Aghajanian et al., 2008). To assess whether discontinuity or absence of junctions were evident post mechanical strain, immunocytochemistry analysis of ZO-1 was performed at 2 h following insult. These studies were also compared with morphological changes, mainly the appearance of stress fibers. As expected in the uninjured control, intense immunopositive staining of the tight junction protein, ZO-1, can be found at the perimeter or junctional areas of every cell (Figure 2). Although ZO-1 is a cytosolic protein, its critical role is to anchor membrane tetraspanin tight junction proteins and thus it appears junctional (Fanning et al., 1998). Notably, uninjured cells have minimal F-actin stress fiber formation, and instead retain more cortical appearing actin. Meanwhile, BMVECs that had undergone strain-elongation had increased F-actin stress fiber formation (red arrows) and ZO-1 was no longer junctional or co-localized with actin. In addition, the normal intense ZO-1 immunostained pattern became punctate and absent following mechanical insult (yellow arrowhead). Therefore, evaluation of docking TJP, ZO-1 showed that TJ complexes are disrupted as a consequence of increased strain.

**Figure 3:**
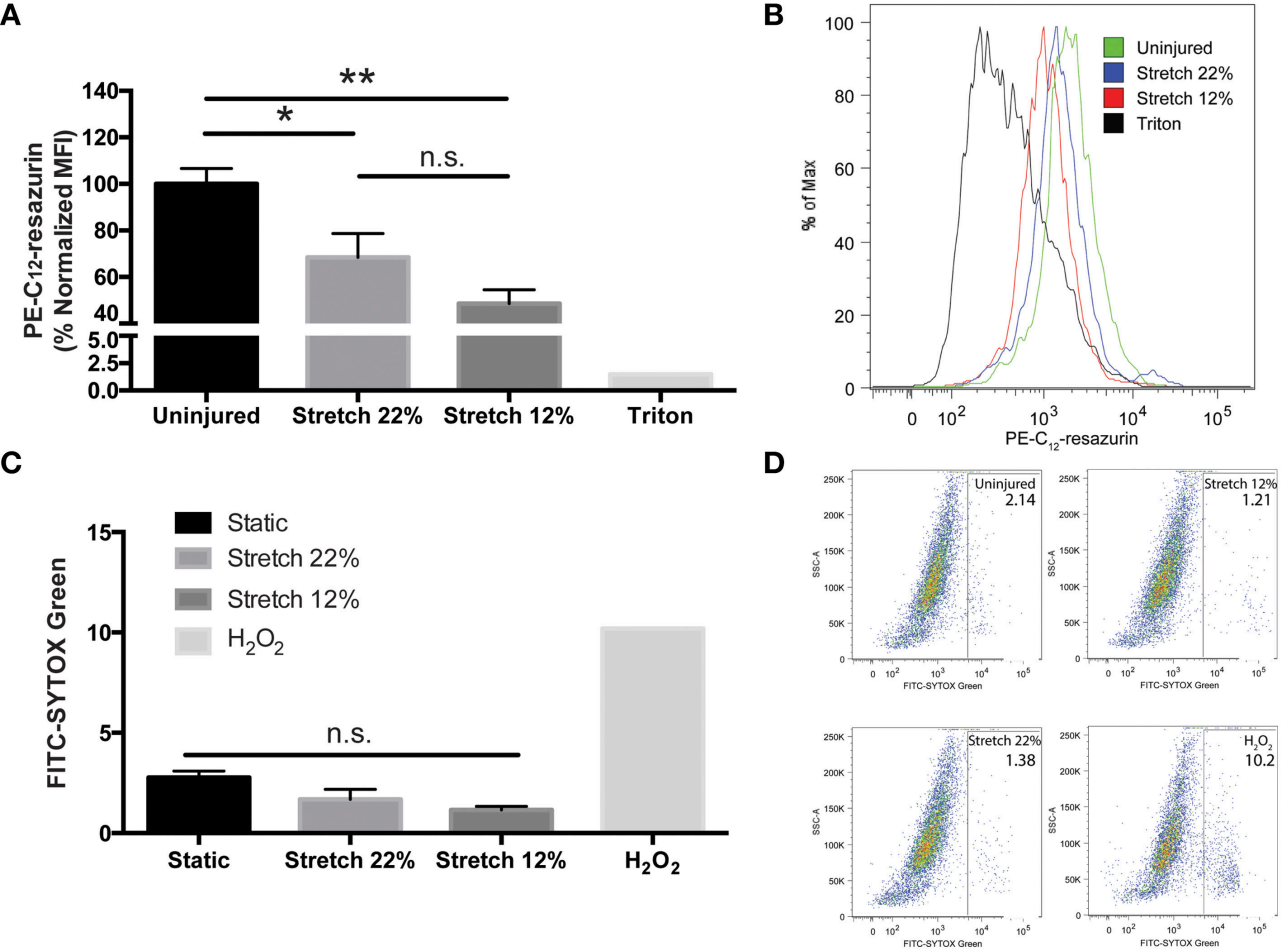
***In vitro* endothelial injury decreases metabolic activity without inducing cell death in BMVECs. (A)** PE mean fluorescence intensity (MFI), a measure of cell metabolic activity, was analyzed in injured and uninjured BMVECs using flow cytometry. Injured cells exhibited reduced red fluorescence intensity reflecting a decrease in metabolic activity, which converts C_12_-resazurin to red-fluorescent C_12_-resorufin. A positive control (0.1% Triton X-100), which disrupts cell membranes and reduces metabolic activity, was used to verify the assay (One-way ANOVA and Tukey *post-hoc* test average ± SEM uninjured *n* = 8, 12% *n* = 5, 22% *n* = 6, ^*^p < 0.05, ^**^p < 0.01). **(B)** Representative histograms of PE-C_12_-resazurin MFI for each condition. **(C)** Cell death was determined using STYOX® Green, a green fluorescent nuclear and chromosome counterstain, was used to determined the percentage of dead cells. A positive control (2 mM H_2_O_2_) was used to induce cell death. (One-way ANOVA and Sidak *post-hoc* test average ± SEM uninjured *n* = 4, 12% *n* = 5, 22% *n* = 6). **(D)** Representative dot plots are shown for each condition.

To determine whether micro-tears and TJ damage resulting from strain induces changes to metabolic activity, BMVECs were evaluated at the acute (24 h) phase of mechanical injury with fluorescence reporter PE-C_12_-resazurin. Analysis of cells by flow cytometry showed that strain decreased metabolic activity as measured by a decrease in the mean fluorescence intensity (MFI) of PE-C_12_-resazurin fluorescence (Figures 3A,B). The results showed a 30 and 50% decrease in mean fluorescence intensity (MFI) for 22 and 12% stretch, respectively when compared to control. Together these results support the notion that in addition to disrupted tight junctions, changes in cellular energetics also occur due to mechanical injury in BMVECs. Of note, analysis of cell death did not show any significant loss of BMVECs following biomechanical injury at any of the points investigated (Figures 3C,D).

### Mechanical injury induces a time-dependent release of eMVs containing TJPs and endothelial markers from brain ECs

**Figure 4:**
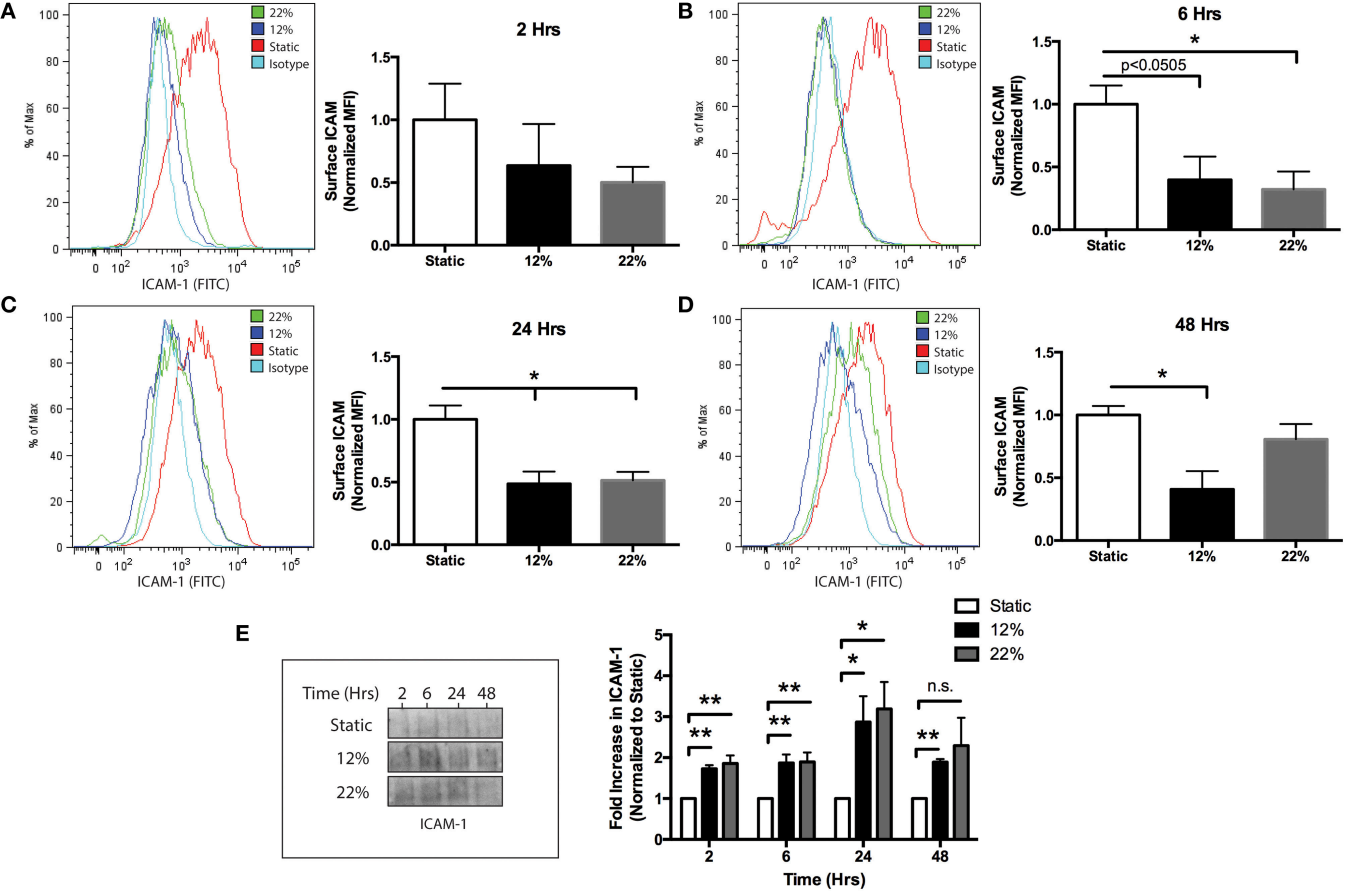
**Reduced surface expression of ICAM-1 and induced production of eMVs containing ICAM-1 following *in vitro* biomechanical injury of brain endothelial cells. (A–D)** show surface ICAM-1 expression by flow cytometry, histograms and bar graph representation for the following time points post injury: **(A)** 2 h, **(B)**, 6 h **(C)**, 24 h **(D)** 48 h (two-tailed *t*-test ^*^p < 0.05 average ± SEM at rest *n* = 4, 12, and 22% stretch *n* = 3 at *t* = 2 and 6 h, *n* = 4 *t* = 24 h, *n* = 6 *t* = 48 h) **(E)** eMVs were collected from the media at the indicated time points and analyzed by western blot. Injured cells released greater quantities of ICAM-1, which was time and stretch dependent. (Two-way ANOVA and students *t*-test ^*^p < 0.05, ^**^p < 0.01, average ± SEM Static *n* = 5, 12% *n* = 4, and 22% *n* = 5 except one outlier was excluded for 12% *t* = 48 h and 22% *t* = 2 h using Grubb's test *p* < 0.05).

**Figure 5:**
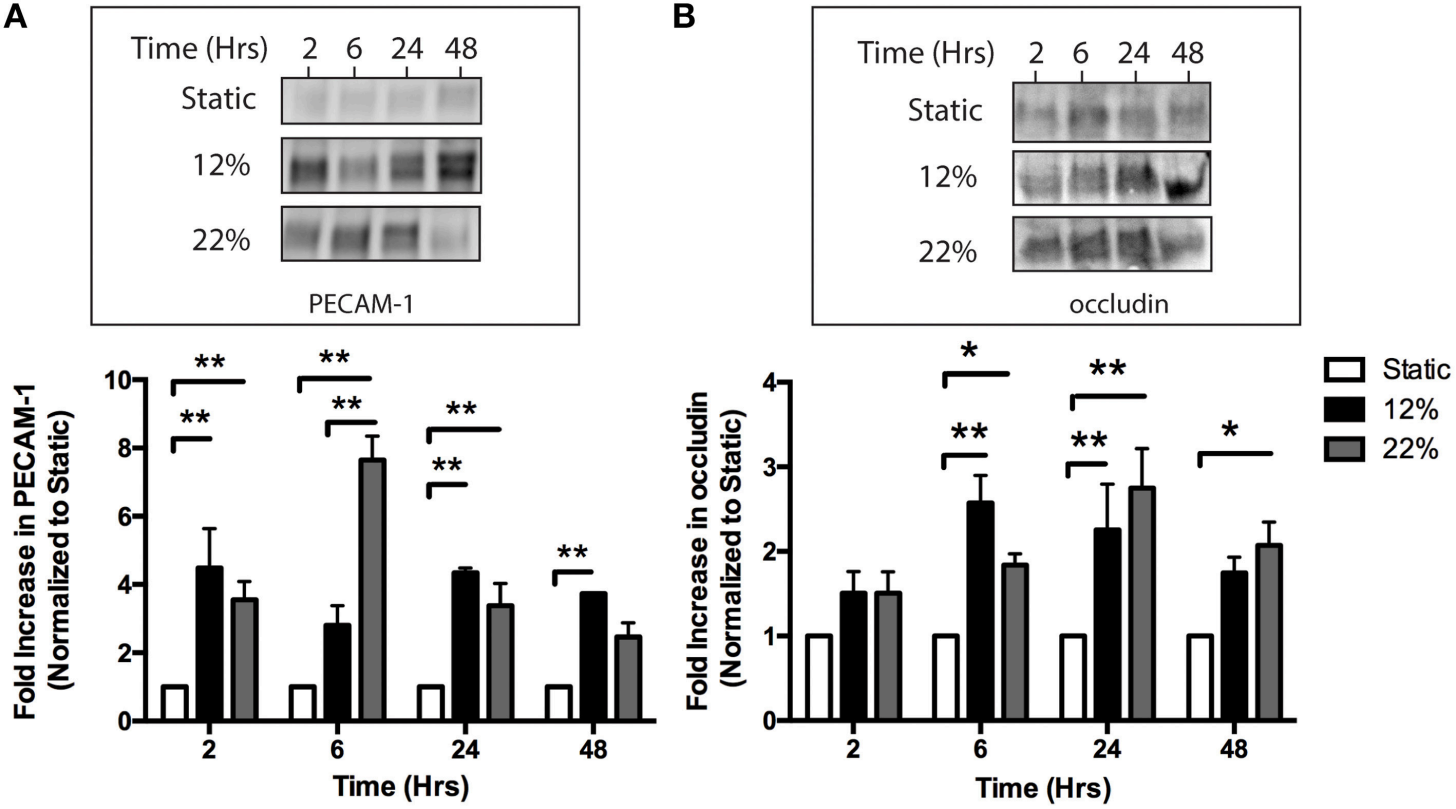
***In vitro* mechanical injury induces the release of eMVs containing the endothelial marker PECAM-1 and tight junction protein occludin. (A)** Injury increases the release of eMVs containing PECAM-1. eMVs were isolated from media using Exoquick and analyzed by western blot. Elevated levels of PECAM-1 were detected at 2 h and remained elevated for most time points. (Two-way ANOVA with Bonferroni *post-hoc* analysis ^**^p < 0.01 average ± SEM Static and 22% *n* = 6, 12% *n* = 4) **(B)** Injury induces a time-dependent increase in eMVs containing occludin. eMVs were isolated from the media using Exoquick at the indicated time points post-injury and analyzed by western blot. Statistically significant increases were seen at 6, 24, and 48 h after injury. (Two-way ANOVA with Bonferroni *post-hoc* analysis ^*^p < 0.05, ^**^p < 0.01 average ± SEM Static and 22% *n* = 6, 12% *n* = 4 except for 12% *t* = 24 h where one outlier was excluded using Grubb's test *p* < 0.05).

To evaluate whether mechanical injury induces the release of eMVs from BMVECs, BMVECs were subjected to the strain parameters characterized earlier (12 and 22% stretch) and cells and media were collected at the times indicated. As an initial aspect of the secondary phase of TBI, endothelial cells upregulate surface expression of ICAM-1. This is thought to occur in order to recruit immune cells to sites of inflammation (Whalen et al., 1999). Interestingly, the overall surface expression of ICAM-1, as determined by flow cytometry, decreased on BMVECs following strain, trending downward as early as 2 h and reaching significance at 6 h. ICAM-1 expression remained decreased at 48 h post strain (Figures 4A–D). This effect on ICAM-1 surface expression followed the same trend for both mechanical insults tested. Conversely, ICAM-1 expression on shed eMVs isolated from the media was significantly increased, thus inversely correlating to the observation in cellular expression (Figure 4E). Further analysis of the isolated eMVs showed the presence of endothelial marker PECAM-1 and tight junction protein occludin following injury (Figures 5A,B). PECAM-1 expression in eMVs was increased rapidly (at 2 h) and persisted at elevated levels (Figure 5A). The increase in occludin, however, was time-dependent and peaked at 24 h post injury (Figure 5B).

The involvement of the small GTP-protein ARF6 in both vascular permeability and microvesicle formation has been recently documented (Muralidharan-Chari et al., 2009; Akers et al., 2013; Ghossoub et al., 2014). Therefore, it was examined whether ARF6 would also play a role in BMVEC eMV shedding following changes in mechanical strain. ARF6 activity was measured using a G-LISA assay that captures ARF6 and then detects ARF6 bound to GTP with another specific antibody. Activation of ARF6 increased significantly by 38% 5 min after the cells were subjected to biaxial strain. Constituently active ARF6 was used as positive control for the assay. (Two-tailed *t*-test average ± SEM Static 0.3362 ± 0.0540, *n* = 6, 22% stretch 0.4644 ± 0.07, *n* = 3 ^*^p < 0.05, positive control 0.7279 ± 0.0787, *n* = 2). The results from this assay point to the possible involvement of ARF6 in eMV biogenesis resulting from kinetic forces acting on brain endothelium.

### Extracellular microvesicles (eMVs) containing occludin are detected in blood plasma following TBI

**Figure 6:**
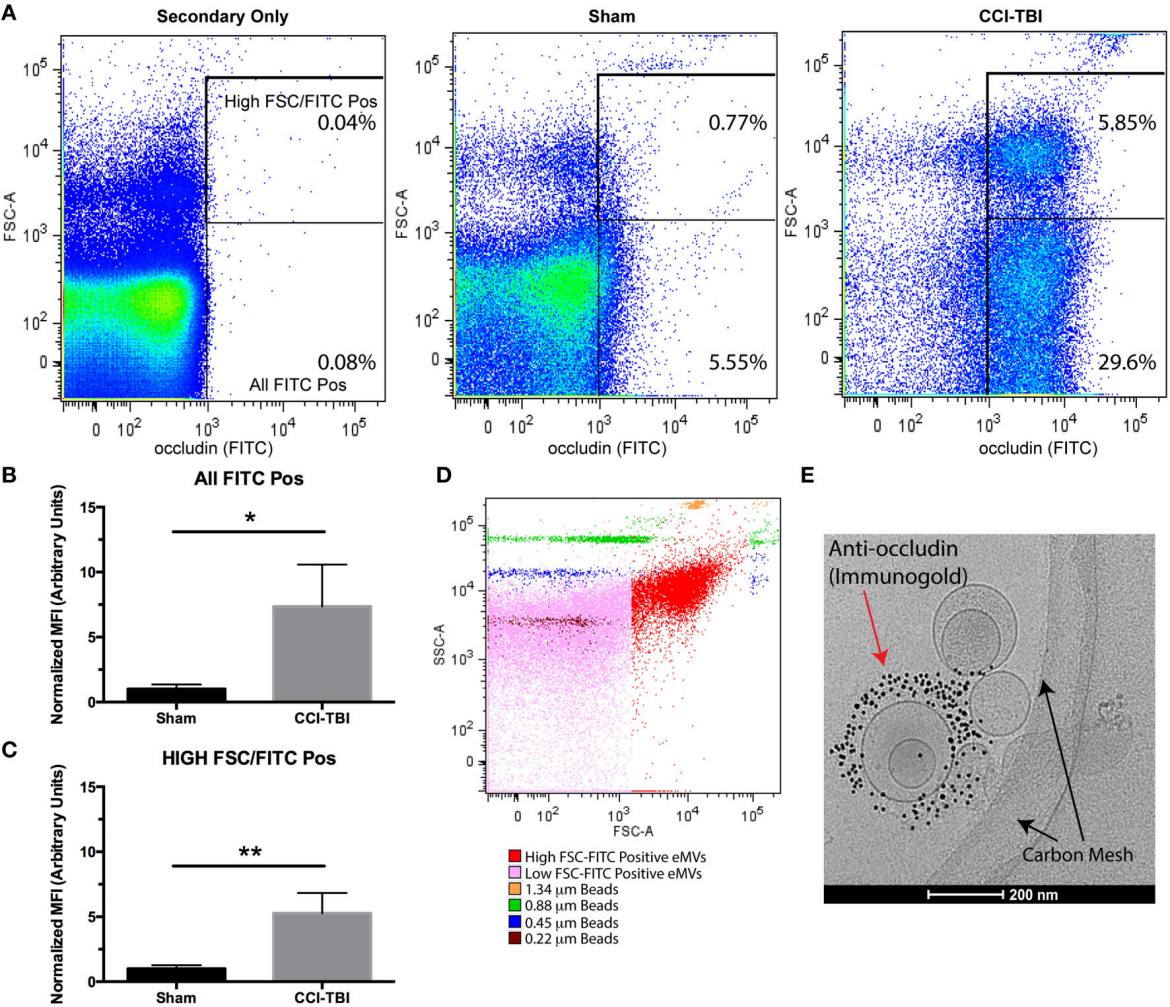
**Experimental TBI induces the release of eMVs containing the tight junction protein occludin**. Mice were subjected to sham surgery or a Controlled Cortical Impact, CCI-TBI and blood was collected 24 h post-injury. **(A)** Flow Cytometry detection of occludin (FITC) positive eMVs from blood plasma. Regions of interest were drawn around all FITC positive vesicles as well as a clear high FSC population. **(B,C)** TBI induces an increase in total FITC positive and high FSC-FITC positive vesicles at 24 h post-injury (Students two-tailed *t*-test average ± SEM *n* = 5 for sham and *n* = 4 for TBI, ^*^p < 0.05, ^**^p < 0.01, one was excluded from the sham group for being a significant outlier). **(D)** Occudin (FITC) positive vesicles (shown in red) were compared to beads of known size ranging from 0.22 to 1.34 μm in diameter. **(E)** eMVs isolated post-TBI were analyzed using cryo-EM and immunogold labeling to show occludin positive microvesicles.

A recent study has indicated that elevated levels of occludin are detectible in the blood of patients following a mild TBI (Shan et al., 2016). Importantly, the above study did not measure occludin in isolated eMVs. To determine if occludin was released in eMVs following TBI, blood plasma was collected 24 h after experimental TBI using the controlled-cortical impact (CCI) mouse model. Purified eMVs were analyzed by flow cytometry and cryo-EM. Figures 6A–E, shows the results from flow-cytometry analysis comparing the levels of occludin positive eMVs detected in blood plasma of sham versus CCI-TBI mice. Two clear occludin positive populations were detected and were designated as either high or low forward side scatter (FSC). FSC is related to the amount of laser passing around the vesicle and is thus related to its size. Analysis of occludin positive vesicles showed a 5-fold increase high-FSC vesicles (Figure 6C) and a 7.5-fold increase in total occludin positive vesicles (Figure 6B) following CCI-TBI as compared to sham. Size beads (ranging from 0.22 to 1.34 μm diameter) were used to estimate the size of occludin positive vesicles (Figure 6D). Occludin positive vesicles appeared to be predominately smaller than 0.45 μm diameter. To further characterize the morphology of eMVs produced following TBI, indirect imaging by high-resolution cryo-EM which has previously been used to visualize extracellular vesicles can provide information regarding eMV size, structure, and composition (Issman et al., 2013). As can be seen in the sample cryo-EM image shown (Figure 6E), eMVs had diameters ranging between 50 and 300 nm. Furthermore, immunogold labeling clearly outlined occludin positive vesicles primarily in the size range of 100–300 nm in diameter (Figure 6E). Of note, not all eMVs in the sample image show immunoreactivity for occludin, indicating the possibility that these eMVs may be from other cellular sources other than brain endothelium. These analyses provide strong *in vivo* evidence for the presence and robust production (at 24 h) of occludin containing eMVs in the blood serum of animals following CCI-TBI. The data also demonstrates for the first time visual confirmation of isolated eMVs highly enriched with the tight junction protein occludin (i.e., as seen by the presence of immunogold antibodies to occludin).

## Discussion

Prior studies have demonstrated the significance of BBB damage following TBI. However, our understanding of the effects of mechanical injury on cerebral vascular endothelium remodeling remains incomplete. We have therefore utilized both *in vitro* and *in vivo* approaches to study responses of the cerebral endothelium to mechanical injury. Here we present compelling results that demonstrate that brain endothelial cells release eMVs containing the TJP occludin in response to biaxial strain (as a means to simulate the primary or kinetic induced injured that is seen in TBI) and the controlled cortical impact animal model of TBI.

Devices that involve deformation of a cellular support membrane (Winston et al., 1989; Schaffer et al., 1994; Brown, 2000) are commonly used to study the effects of physiological strain on endothelial cells (Vion et al., 2013a; Figueroa et al., 2014) and TBI-like injury on neurons and glial cells (Ellis et al., 1995; Geddes-Klein et al., 2006; Cullen et al., 2007). Only recently have these devices been used to model the effects of TBI on endothelial cells (Salvador et al., 2013, 2015). Characterization of the injury induced from the *in vitro* mechanical strain model used in this study reproduces the type of linear kinetic forces that induces micro-tears to the endothelium in TBI (Figure 1). Indeed we also observed that mechanical injury induced changes in metabolism resulting from membrane damage (Figures 3A,B). While not significantly significant, there was a trend for a greater decrease in metabolic activity from 12% substrate elongation as compared to 22% elongation. It is unclear if this is a result of small differences in the membrane elongation profile achieved using our experimental set-up or if it is a real endothelial physiological response to mechanical injury. Furthermore, stretch injury models such as these typically do not increase cell death even on neuronal cells subjected to strain/stretch ranging from 0 to 50% (Geddes-Klein et al., 2006). We also saw no increase in cell death following substrate elongation (Figures 3C,D). Instead, many endothelial cells appeared to be swollen following injury (Figure 1), which has been reported by others both *in vitro* (Salvador et al., 2013, 2015) and *in vivo* (Simard et al., 2010). This change in cell volume is theorized to alter cytoskeletal dynamics and weaken tight junctions leading to the increased barrier permeability (Simard et al., 2010).

Short-term studies (24–72 h) have found that *in vitro* and *in vivo* models of TBI decrease endothelial expression of TJPs (occludin, ZO-1, and Cld-5; Higashida et al., 2011; Hue et al., 2013; Wen et al., 2014; Salvador et al., 2015) and result in discontinuous ZO-1 staining in histological analysis of brain tissue (Thal et al., 2012). We also observed disrupted tight junctions and a transition of ZO-1 expression from continuous localization along cell borders to a discontinuous, punctate, and even absent expression pattern (Figure 2). These changes in ZO-1 localization and subcellular distribution were accompanied by decreased colocalization with F-actin (Figure 2). The colocalization of F-actin with TJPs has been well documented, and actin has been proposed to play a critical role in maintaining TJ complex integrity and stability (Lai et al., 2005). While TJP disassembly and expression changes have been reported to occur following TBI, it has been unclear whether TJPs are degraded internally or processed and packaged to be released extracellularly. Here we present for the first time evidence that both *in vitro*, via mechanically induced strain (Figure 5), and *in vivo*, using a leading model of experimental TBI (Figure 6), mechanical injury increases the release of eMVs containing the TJP occludin.

The endothelium is constantly exposed to mechanical forces such as shear stress and strain and consequently remodels in response to these mechanical forces. The production of eMVs by endothelial cells following mechanical stimuli has only recently been investigated in the context of physiological shear stress and cyclic strain *in vitro* (Vion et al., 2013a,b; Letsiou et al., 2015) and *in vivo* (Jenkins et al., 2013). Other studies have shown that human pulmonary and umbilical endothelial cells (HUVECs) respond to mechanical forces to produce microparticles and that the mechanism of cyclic stretch-induced vesicle release is thought to be activated by the release of TNF-α and other cytokines (Wang et al., 2003; Dignat-George and Boulanger, 2011). A recent study using an *in vitro* TBI model on endothelial cells showed an increase in TNF-α and CCL2 mRNA production 24 h post injury (Salvador et al., 2015). TNF-α and other inflammatory cytokines are known to induce the upregulation of ICAM-1 in brain ECs (Ramirez et al., 2012; Rom et al., 2015). Therefore, we anticipated that ICAM-1 surface expression would be upregulated following mechanical injury. However, we observed a decrease in ICAM-1 surface staining, which corresponded to an increase in shedding of ICAM-1 in eMVs (Figure 4). Upregulation of ICAM-1 and other adhesion molecules by the cerebral endothelium following a TBI is well established (Carlos et al., 1997). An important distinction between our work and previous studies utilizing *in vitro* TBI models is that our data reflects changes in primary human BMVECs vs. an immortalized murine brain ECs line (Salvador et al., 2013, 2015). In addition, Salvador et al. (2015) detected increased mRNA for inflammatory cytokines but they did not evaluate other indices of endothelial activation such as cytokine release or protein expression of adhesion molecules (Salvador et al., 2015). The lack of ICAM-1 induction in brain ECs has led us to hypothesize that in the absence of other neurovascular unit (NVU) members (i.e., microglia, astrocytes), brain ECs likely initiate repair or angiogenic mechanisms without activation. This is supported by studies showing the production of by cytokines produced by other cells of the NVU (Carlos et al., 1997), a contribution which is lacking in our *in vitro* experimental model. Therefore, as basal cellular surface levels of ICAM-1 decrease via shedding of eMVs, brain ECs may only upregulate ICAM-1 in response to signals from other injured cells (i.e., microglia, astrocytes, and neurons) or by recruited immune cells. In summary, increased eMV production in response to mechanical injury was observed in both *in vitro* and *in vivo* models and represents an understudied aspect of the consequences of TBI.

The profile of ICAM-1 release in eMVs was similar at both 12 and 22% elongation while the profile of PECAM-1 and occludin had differential changes. Specifically, at 12% elongation, PECAM-1 release remained relatively constant at all time points while 22% elongation induced a graded release that peaked at 6 h. Occludin expression in eMVs was also a graded response, which peaked earlier in response to 12% as compared to 22% elongation. While it might be expected that eMV release would be linear in response to mechanical injury, where greater injury induces greater release for all proteins, little is known regarding this process. Our results provides some initial insight into the response of brain ECs to strain *in vitro* and *in vivo*, and further investigation would provide a more thorough understand of the kinetics of eMV release from mechanical injury.

Regarding extracellular vesicles, two main categories have been identified based on their size and mechanism of release. Microparticles (MPs) are the larger of the two categories ranging between 0.1 and 1 μm in diameter and are generated by outward blebbing of the plasma membrane. Exosomes are smaller (30–100 nm) and are released by vesicular fusion with the plasma membrane (Raposo and Stoorvogel, 2013). In this study we have not focused on identifying the type of extracellular vesicle that contains the TJP occludin or endothelial markers (PECAM-1, ICAM-1). A number of studies have reported on the localization of both PECAM-1 and ICAM-1 in MPs (Jimenez et al., 2003). Our flow cytometry data of occludin positive eMVs from blood plasma (Figure 6A) identified two clear populations based on size. However, standard flow cytometers do not have the resolution to accurately identify exosomes based on their small size. Thus it is unclear if the two populations reported by flow cytometry here translate to the two types of vesicles. Occludin-immunogold labeling and cryo-EM imaging (Figure 6E) showed occludin positive vesicles that fall into the size category of both types of extracellular vesicles. Aside from identifying occludin positive vesicles, a number of vesicles detected by these methods appeared to be multivesicular which has been shown previously (Issman et al., 2013). Of note, the detection of immunogold labeled vesicles that appeared to be inside another vesicle likely reflects two vesicles stacked on top of one another (arrow) instead of a multivesicular vesicle (arrowhead). In addition, some vesicles appeared to be more granular than others which suggests differences in content (Issman et al., 2013).

The mechanism of extracellular vesicle release has been linked to cytoskeletal rearrangement (Boulanger et al., 2006; Latham et al., 2013) as well as small GTPases such as Rho (Burger et al., 2011) and ARF6 (Pasquier et al., 2014). ARF6 has been linked to both microvesicles (Pasquier et al., 2014) and exosome release (Friand et al., 2015). In the case of microvesicles, ARF6 activation leads to downstream phospholipase D and ERK signaling, MLC activation and finally acto-myosin contraction at the vesicle neck and the release into the extracellular space (Muralidharan-Chari et al., 2009). For exosomes, ARF6 and phospholipase D2 control the budding of intraluminal vesicles into multivesicular bodies. Multivesicular bodies will later fuse with the membrane to release the exosomes extracellularly (Ghossoub et al., 2014). Based on the evidence for ARF6 involvement in extracellular vesicle release we analyzed ARF6 activation and found that it was elevated following injury (Results Section). The activation of ARF6 and detection of eMVs following injury contributes to our understanding of the effects of mechanical damage to the endothelium.

We believe that the results presented here represent a significant advancement to our understanding of cerebral vascular remodeling following TBI. In addition, our approach to understanding the effects of TBI is highly BBB and endothelial centric and offers the potential to uncover new biomarkers for the monitoring and diagnosis of TBI. The field of TBI research has indeed recognized the lack of available effective biomarkers for diagnosing and monitoring a patient following brain injury (Forde et al., 2014), and many prior studies have focused on biomarkers related to neuronal and astrocytic damage with few focused on biomarkers specific for BBB damage. Cardiovascular research has spearheaded studies focusing specifically on endothelial damage and the production of extracellular vesicles as a diagnostic and prognostic tool for hypertension, atherosclerosis, diabetes, and endothelial dysfunction (Boulanger et al., 2006; Chironi et al., 2009). Numerous studies have shown that endothelial cells (pulmonary, umbilical, artery, aortic) release eMVs in response to a number of insults including inflammatory cytokines, mechanical injury, and serum deprivation (Chironi et al., 2009). In these studies, eMV levels are typically determined by Annexin V (microparticles; Draeger et al., 2011) or exosome markers (CD63, Alix, etc.), which are general markers and are therefore not cell or organ specific. Some studies have proposed the differentiation of the cell lineage by cell specific markers such as P-selectin (CD62P) and CD63 for platelets (Tan et al., 2005) and PECAM-1 (CD31) for endothelial cells (Boulanger et al., 2006). Here, we propose an alternative method of eMV detection and identification by TJPs. Coupled with endothelial markers and other proteins enriched at the BBB, this approach delineates eMV origin, primarily from brain ECs, and also has the potential to reflect the functionality of the host cell and provide a clinically useful assessment of BBB status in TBI. Detection of brain endothelial-derived eMVs could provide a novel means to analytically evaluate BBB health in a number of CNS conditions that are associated with trauma and neuroinflammation.

## Author contributions

AA and SR contributed to the study design. AA, EL, SM conducted the experiments. AA, SR, EL, SM, RR contributed to the writing of the manuscript.

### Conflict of interest statement

The authors declare that the research was conducted in the absence of any commercial or financial relationships that could be construed as a potential conflict of interest. SR is an inventor in a US patent application related to brain endothelial-derived extracellular microvesicles. The US patent application number is filled under US 14/406,400; PCT/US2013/047470 and is entitled “Method for detecting injury to the brain”. The application priority date was 6/26/12, filling date 6/26/12 and publication date 5/21/2015.
